# Crystal Structure of Exotoxin A from *Aeromonas* Pathogenic Species

**DOI:** 10.3390/toxins12060397

**Published:** 2020-06-15

**Authors:** Geoffrey Masuyer

**Affiliations:** Department of Pharmacy and Pharmacology, Centre for Therapeutic Innovation, University of Bath, Bath BA2 7AY, UK; gm283@bath.ac.uk

**Keywords:** ADP-ribosyltransferases, exotoxin, bacterial toxin, X-ray crystal structure, *Aeromonas*

## Abstract

*Aeromonas* exotoxin A (AE) is a bacterial virulence factor recently discovered in a clinical case of necrotising fasciitis caused by the flesh-eating *Aeromonas hydrophila*. Here, database mining shows that AE is present in the genome of several emerging *Aeromonas* pathogenic species. The X-ray crystal structure of AE was solved at 2.3 Å and presents all the hallmarks common to diphthamide-specific mono-ADP-ribosylating toxins, suggesting AE is a fourth member of this family alongside the diphtheria toxin, *Pseudomonas* exotoxin A and cholix. Structural homology indicates AE may use a similar mechanism of cytotoxicity that targets eukaryotic elongation factor 2 and thus inhibition of protein synthesis. The structure of AE also highlights unique features including a metal binding site, and a negatively charged cleft that could play a role in interdomain interactions and may affect toxicity. This study raises new opportunities to engineer alternative toxin-based molecules with pharmaceutical potential.

## 1. Introduction

Members of the diphthamide-specific class of mono-ADP-ribosyl transferases (mART), which include the diphtheria toxin (DT), *Pseudomonas* exotoxin A (PE) and *Vibrio cholerae* cholix (Chx) [1], are potent bacterial toxins that specifically modify the diphthamide residue of eukaryotic ribosomal elongation factor 2 (eEF2), which plays an essential role in protein synthesis [2]. These toxins catalyse the transfer of ADP-ribose from nicotinamide adenine dinucleotide (NAD^+^) onto the diphthamide, which results in inhibition of protein synthesis and thus death of the host eukaryotic cells [3].

PE and cholix share a similar structure and mechanism of action. They consist of 66-68 kDa A-B type toxins arranged in three functional domains (I-III) that support receptor binding, translocation and catalytic activity, respectively. In order to reach its cytosolic destination, the toxins first bind to LRP1 (low density lipoprotein receptor-related protein 1) at the surface of target cells, which is followed by receptor-mediated endocytosis [4,5]. The toxins are then activated by the furin protease into di-chain molecules and are retrogradely transported to the endoplasmic reticulum (ER). The furin-generated carboxy-terminal fragment, which includes the enzymatic domain, is next translocated to the cytosol [6], where it can ADP-ribosylate eEF2 [7].

The potent cytotoxicity of mART toxins has been exploited therapeutically as part of immunotoxins. These engineered molecules consist of a toxin catalytic fragment devoid of its endogenous cell-binding domain but combined with an antibody or a cytokine that redirects toxicity towards a specific cell type [8]. PE is for example the active component of Moxetumomab Pasudotox (Lumoxiti), an immunotoxin recently approved by the FDA for the treatment of hairy cell leukaemia [9]. Other applications developed from the toxins include vectors for mucosal delivery of vaccines [10] or oral delivery of biological drug products [11], which in both cases utilise the intrinsic ability of the toxins to rapidly and efficiently move across intact epithelia [12]. The discovery of additional mART toxins should therefore provide useful alternative tools for the design of toxin-based pharmaceuticals with unique pharmacological properties.

A recent study, reporting on a clinical case of necrotising fasciitis caused by *Aeromonas hydrophila* infection, identified a PE-like exotoxin from functional genomics analysis [13]. This *Aeromonas* exotoxin, AE, was later shown to be one of the main virulence factors promoting pathogenesis of the polymicrobial infection [14]. *Aeromonas* are Gram-negative, facultative anaerobic bacteria, commonly found in aquatic environments [15]. An increased resistance to water treatment and antibiotics [16] has made *Aeromonas* species into emerging human pathogens, particularly in areas hit by natural disasters such as hurricanes and tsunamis [17]. They are responsible for a wide range of human diseases that include intestinal and extraintestinal pathologies and are most often associated with acute gastroenteritis, skin and soft tissue infections but have also been observed to cause more systemic conditions such as septicaemia and meningitis [15,17]. Aeromonas also causes haemorrhagic septicaemia in fish, which is associated with high mortality and thus severe economic losses in aquacultures [18]. *Aeromonas* pathogenesis is promoted by an arsenal of virulence factors including several exotoxins, such as the cytotoxic enterotoxins Act and aerolysin, as well as extracellular enzymes and secretion systems [19].

Here AE is shown to be conserved across several pathogenic *Aeromonas* species. The crystal structure of AE was solved at 2.3 Å and presents a three-domain fold similar to other diphthamide-specific mART toxins. The homology with PE at key structural elements suggests that AE follows a canonical mechanism of action that ends in ADP-ribosylation of eEF2. The structure of AE offers a unique framework to design novel compounds against diseases caused by *Aeromonas* and provides a new tool for the design of toxin-based therapeutics.

## 2. Results and Discussion

### 2.1. Genomic Data Mining Suggests AE is Common to Several Aeromonas Species

The NCBI database was screened for exotoxin A homologues in *Aeromonas*. Using PSI-BLAST, a total of 51 protein sequences were found that displayed at least 60.3% homology to PE (UniProtKB - P11439) with an E-value below 0.001 and also confirms previous findings by Grim et al. [13]. The dataset includes exotoxin A from several species including *A. hydrophila* and *A*. *dhakensis,* which share over ≥ 95.5% identity across 40 entries (Supplementary Material: Figures S1 and S2). An additional closely related cluster with ≥ 85% identity was also found in *A. salmonicida*, *A. piscicola*, and *A. bestiarum*. All of these species are associated with human or zoonotic diseases [20].

**Figure 1:**
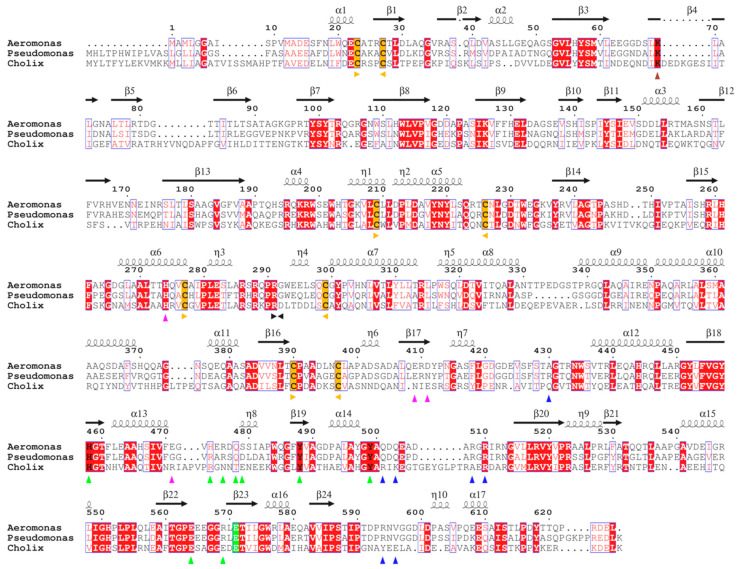
Aeromonas exotoxin sequence analysis. Sequence alignment of *Aeromonas* exotoxin A with *Pseudomonas* exotoxin A (PE) and Cholix. Conserved cysteines are highlighted in yellow, strictly identical residues with a red background, and similar residues with red characters. Figure prepared with ESPript [21]. Important residues based on similarity with PE are marked with triangles: furin cleavage site (positions 291–292) in black, receptor binding site in red, eEF2 binding site in green, nicotinamide adenine dinucleotide (NAD^+^) binding and catalytic residues in blue; *Aeromonas* exotoxin A (AE) metal ion coordination in pink. Secondary structure elements of AE are presented above the sequence.

At the primary sequence level, AE presents all the features of other mART toxins [21,22]. It is 626 residue-long (67 kDa), and pairwise sequence alignment with PE and Cholix (Figure 1) shows conservation of key cysteines and of the furin cleavage site (RQPR), which is responsible for activation of the toxin and release of the catalytic fragment [23]. AE also possesses a KDEL-like C-terminal sequence (RDEL) that in PE was shown to be necessary for retrograde transport to the endoplasmic reticulum of intoxicated cells [24].

Interestingly, sequence comparison with PE and Cholix shows that homology varies across the length of the toxin (Table 1). Domain Ia (residues 15–263, all AE sequence numbering are based on the full-length toxin), which is responsible for receptor-binding, is the least conserved. On the other hand, domain III (residues 414–626), which holds the catalytic activity, shows the highest sequence identity to PE (71.9%) and Cholix (45.8%).

Recombinant AE produced here was a catalytically inactive mutant E571A, corresponding to residues 13–626 of the full-length toxin. A potential N-terminal leader peptide sequence (1–11) was omitted, which in PE corresponds to the signal peptide (1–25) cleaved from the toxin precursor during secretion. Instead, AE was expressed and purified with a N-terminal poly-histidine tag and successfully crystallised.

### 2.2. Crystal Structure of AE

AE crystallised in the P12_1_1 space group (Table 2) with two identical molecules per asymmetric unit (rmsd of 0.62Å over 601 Cα atoms). Residues 15–622 were present, with gap in the electron density map observed for segment 228–230, as well as the missing N- (tag) and C-termini (623–626), all in solvent-accessible areas.

**Figure 2:**
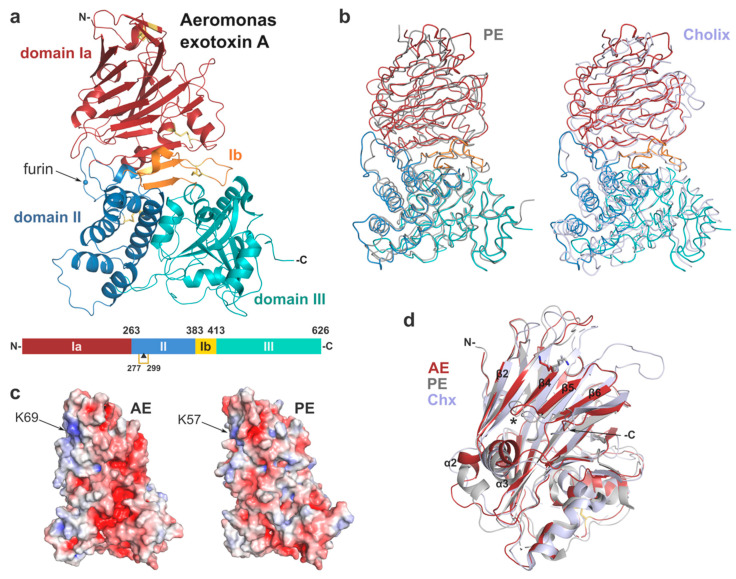
Crystal structure of Aeromonas exotoxin A. (**a**) Overall structure with domain Ia (15–263), red; domain II (264–383), blue; domain Ib (384–413), orange; and domain III (444–626), cyan. Disulphide bridges are shown as yellow sticks, and the furin cleavage site is marked by a blue sphere. Schematic drawing of AE; the disulphide bridge between cysteines 277 and 299 linking the two chains after furin cleavage (▲) is shown in gold. (**b**) Superposition of AE (colour as above) onto the structure of PE (left, grey, PDB 1IKQ) and cholix (right, light blue, PDB 2Q5T). (**c**) Electrostatic surface potential of AE and PE calculated using the APBS tool in PyMOL (scale from −5, blue to + 5, red). AE in the same orientation as (a); arrows mark the location of residue K57 involved in PE binding to the LRP1 receptor [4], and its equivalent in AE (K69). (**d**) Superposition of domain I only from AE (red), PE (grey) and cholix (light blue), with lysine described above in stick representation. Important secondary structure elements of AE are labelled, with star indicating the PE loop (position 60) involved in receptor-binding [26,27].

Overall, the structure of AE presents a tri-domain architecture similar to PE and cholix (Figure 2, Table 1) with root-mean-square deviation (rmsd) of 2.5 and 2.9 Å, respectively, and includes 4 strictly conserved disulphide bridges. Individually the domains show high structural homology with their PE counterpart (rmsd < 2 Å). Domain I consists of a core 13-stranded β-jellyroll fold. It is complemented by domain Ib (residues 384–413) that provides two additional β-strands, which run anti-parallel to the β-jellyroll fold and sit at the interface between the three domains. Domain II (residues 264–383), labelled as the translocation domain, presents a compact six α-helices bundle. The catalytic domain (domain III) shows an α/β topology, distinct from typical nucleotide binding folds.

#### 2.2.1. Domain I and Implications for Receptor Binding

The receptor binding-domain presents a β-jellyroll fold, which is reminiscent of lectin-like proteins. A structural search through the PDB database with DALI [28] confirms a distant structural homology (*Z* score ≤ 8.0) to bacterial sugar-binding proteins and human galectins; however, there is no evidence to date of domain I binding to carbohydrates. The main differences between domain I of AE and its homologues are in flexible, surface-accessible linker regions either side of the core β-sheets. In particular, helix α2 is shorter in AE and positioned in continuity to the β2 strand, compared to PE where it runs perpendicular to β2, whilst it corresponds to a simple coil in cholix (Figure 2d).

Another stand out difference are two extended loops in cholix that are not seen in AE and PE between strands β3-β4 and β5-β6, respectively. Remarkably, β4 is the central element of a slightly concave, open surface, which was shown to be implicated in binding to the LRP1 receptor in PE. This strand is particularly well conserved in AE with K69 superposing directly onto K57 (Figure 2c,d). In PE, mutation at this position to glutamic acid caused a 100-fold reduction of toxicity toward mouse fibroblasts [4]. In addition, insertion of a dipeptide (Glu-Phe) at position 60 also showed a 500-fold decrease in cytotoxicity that was associated with disruption of receptor binding [26,27]. Although cholix was shown to recognise LRP1 as well [5], the amino acid sequence of β4 is not conserved, and the key lysine is there occupied by isoleucine (I64), implying a different mechanism of LRP1-binding between PE and cholix. Here, the strong structural homology with PE suggests that AE may recognise and interact similarly with LRP1. However, further experimental work is required to confirm the role of LRP1 or identify other potential cell surface receptors for AE.

#### 2.2.2. Structural Elements Involved in Intracellular Trafficking

Domain II (residues 264–383) presents a six α-helices bundle similar to that in PE, and which was shown to be involved in translocation of the toxin across membranes [29,30], although the mechanism on how this occurs is not fully understood. Importantly, this domain holds a furin protease recognition site identical to the one in PE, corresponding to sequence RQPR (288–291) [23,31], with the scissile bond between R291 and G292. This site resides on a well-ordered loop, which protrudes from domain II and is remarkably accessible at the surface of AE (Figure 2a). Furin cleavage of PE occurs at acidic pH in vitro, most likely reflecting the endosome conditions (pH < 5.5) where cleavage is believed to occur in vivo [31]. Endoproteolytic activation of the toxin into a di-chain fragment is necessary for toxicity [6]. In AE, furin cleavage would result in a carboxy-terminal fragment of 36kDa that holds the catalytic domain and remains associated with the rest of the toxin via a conserved disulphide bridge between C277 and C299. This cysteine bond may be reduced downstream of the endocytic pathway with the help of protein disulphide-isomerase (PID) [32]. AE is expected to follow a similar intracellular route to PE and cholix through the endocytic pathway where it may be activated by furin and trafficked to the Golgi [33]. In the Golgi, PE interacts with KDEL receptors via binding of its REDL C-terminal signal sequence, which results in the toxin’s retrograde transport to the ER [24].

Importantly, PE first needs to be processed in the early stage of intoxication so that its carboxy terminal lysine (REDLK) is removed by an extracellular carboxypeptidase that reveals the REDL signal sequence [34]. Although not optimal for recognition by KDEL receptors, this variant of the canonical sequence is enough to bring PE in the ER [35]. AE’s carboxy terminal end, RDEL, does not contain a final lysine and is also closer in sequence to the preferred KDEL motif (Figure 1). Remarkably, chimeric immunotoxins of PE with a mutated RDEL C-terminal sequence showed up to a 100-fold increase in cytotoxicity compared to the native REDL sequence, which was linked to a stronger affinity for KDEL receptors [36]. These significant sequence differences with PE suggest that AE might be adapted to a more efficient intracellular trafficking. Further work is however necessary to confirm if this translates into higher toxicity.

In the ER, the furin-cleaved toxin fragment undergoes partial unfolding and is exported to the cytosol by retro-translocation. It has been suggested that PE exploits the endoplasmic-reticulum-associated protein degradation (ERAD) system, which involves the Sec61 translocon [37], but manages to avoid proteasomal degradation thanks to its low lysine content that averts poly-ubiquitination [38]. Noticeably, AE is devoid of any lysine in its 36 kDa active fragment, which also supports this hypothesis.

As it reaches the cytosol, it is presumed that the toxic fragment is refolded with the help of host chaperones, such as Hsp90 and Hsc70, as seen with the cholera toxin [39]. A recent study showed that Hsp90 could recognise a RPPDEI-like motif common to several ADP-ribosylating toxins [40], which includes the C-terminal PE sequence (PPREDL). However, the proposed Hsp90 recognition motif requires a dual proline, which is not present in AE, and varies significantly in cholix (Figure 1), suggesting both toxins may use an alternative refolding mechanism.

#### 2.2.3. Domain III and Implications for ADP-Ribosylating Activity on eEF2

Once in the cytosol, the active fragment should exert its activity on eukaryotic elongation factor 2, an essential component of the protein synthesis machinery that promotes translocation of the mRNA and peptidyl-tRNA, and thus movement of nascent polypeptide chains on the ribosome [41]. The molecular target of diphtheria-like toxins is diphthamide, a unique post-translationally modified histidine, common to eukaryotes and archaea.

**Figure 3:**
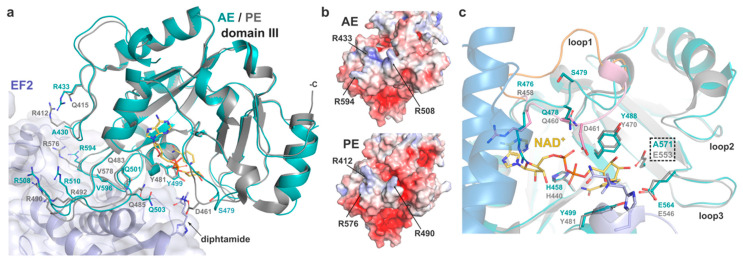
Catalytic domain of AE. (**a**) AE domain III (cyan) superposed onto the structure of PE (grey) in its complex (PDB 2ZIT) with eEF2 (purple) and NAD^+^ (yellow). Residues involved in the eEF2-PE interaction (and AE equivalent) and the target diphthamide are shown as sticks. AE domain I and II were omitted for clarity. (**b**) Surface potential of AE and PE at the substrate binding site (as per 2c). (**c**) Close-up view of the active site with residues involved in NAD^+^-binding and catalysis shown as sticks. Loop 1 from PE in its open conformation (yellow, PDB 1ZM3) is superposed onto the complex where it is in a closed conformation (pink). Position of the catalytic mutant E571A is highlighted. AE domain II is shown in blue.

In PE, domain III is responsible for eEF2 binding and presentation of the catalytic pocket, which holds the NAD^+^ cofactor, towards the target diphthamide. Jørgensen et al. showed that the PE-eEF2 interface mostly involves hydrogen bonds and is fairly malleable, as illustrated by the variation in the toxin positions across several complexes within the same crystal structure [3,7]. Residues involved in PE binding to eEF2 are particularly conserved in AE and overlap remarkably well when comparing the structure of AE with PE in the eEF2-bound complex (Figure 3).

Despite their apparent flexibility, the main carbon chain of AE loops 426–434, 501–513, and 591–600 superposes well with the corresponding PE loops involved in substrate binding (408–416, 483–495, 573–582). In the AE crystal structure, these loops are stabilised by symmetry-related contacts. Sequence comparison shows a small difference where R412 of PE corresponds to A430 in AE. However, the electrostatic potential of the exposed substrate-binding surface is very similar in both toxins, with AE compensating the negative charge with the side chain of R433 instead (Figure 3b). In view of the high primary sequence homology and the adaptable structure of loops and mobile side chains (R/Q) involved in hydrogen and electrostatic bonds that make most of the PE-eEF2 interaction, it is possible that AE may follow a similar strategy of binding to eEF2.

#### 2.2.4. Catalytic Site

The mechanisms of ADP-ribosylation by PE and its homologues have been described in detail previously [2,42]. In AE, the catalytic pocket is composed of the strictly conserved residues E571, H458, Y499 and Y488. Superposition with the structure of PE shows the active site of the two toxins align perfectly (Figure 3c). In PE, the glutamic acid forms a hydrogen bond with the N-ribose moiety of NAD^+^ and orientates the dinucleotide substrate for nucleophilic attack by the diphthamide residue, resulting in ADP-ribosyl-eEF2 and a free nicotinamide [7].

Access of the NAD^+^ cofactor to the catalytic pocket is key to the enzymatic activity and is regulated by movement of three surrounding loops upon binding (loop 1: 475–482, loop 2: 535–539, loop 3: 564–569) that were observed to be flexible across several PE structures [3]. Loop 1 in particular was shown to flip from open to closed conformation in PE, with residues R458 and Q460 being important for substrate docking by making van der Waals interactions and hydrogen bond with the adenine-phosphate of NAD+, respectively. The closed conformation of loop 1 is further stabilised by hydrogen-bonding of D461 with the diphthamide. In the substrate-free AE structure presented here, loop 1 takes on an intermediate position (Figure 3c), which sees the main chain of R476 and Q478 in relatively similar location to their equivalent in the NAD^+^-bound PE structure, with the glutamine side group within hydrogen bond distance of the adenine-phosphate. Although R476 is seen to partially occlude NAD^+^ here, its position is restricted by the presence of domain II. Activation of PE by furin is essential for its enzymatic activity, and it has been suggested that the residual fragment undergoes significant conformational changes that allow for NAD^+^ binding [43]. Loop 1 and R476 should therefore be flexible enough to accommodate substrate binding in activated AE. The main difference in loop 1 is at position 479, where serine is here facing outward. This position was shown to be less critical in PE as mutation D461A did not affect activity [3]. However, the inherent plasticity of loop 1 should allow for S479 to switch back inward upon NAD^+^ binding and make hydrogen bond with the diphthamide amide group, as seen with D461 of PE. On the other side of the pocket, loop 3 is strictly conserved between AE and PE and holds residues E564 and R569 that were shown to be essential for the ADP-ribosyltransferase activity of PE. Again, the structural similarity with PE at the catalytic site, suggests that AE likely follows the same enzymatic mechanism.

#### 2.2.5. Interdomain Interactions

Previous in vitro studies showed that PE undergoes significant conformational changes at acidic pH [44], which also results in presentation of the furin cleavage site [23]. The mechanism behind pH-mediated structural alterations is not evident, but a global protonation effect on charged residues at the interdomain interfaces, involving several electrostatic interactions, may be involved [43]. Beyond the common arginine-rich loop holding the furin site, the interface between the three domains varies significantly between AE and its homologues.

**Figure 4:**
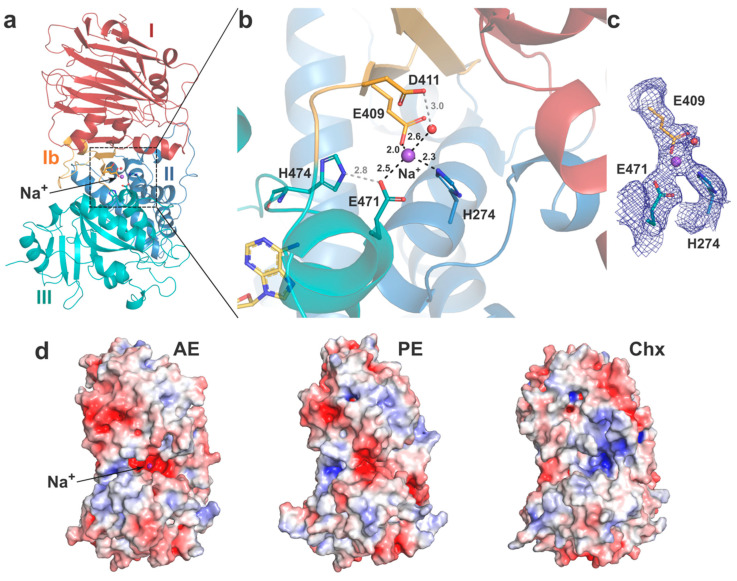
Interdomain interactions. (**a**) Structure of AE (coloured as previously) with the metal ion (Na^+^) shown as a purple sphere. The coordinating residues and water are shown as sticks and red sphere, respectively. (**b**) Close up view of the metal binding site, with coordinating bonds in black dashes and secondary hydrogen interactions in grey, distances labelled in Å. NAD^+^ (yellow) from the PE-bound structure is shown for illustration. (**c**) Electron density map (*2Fo-Fc* map at 1.0σ). (**d**) Electrostatic surface potential of AE, PE and cholix (as per Figure 2c), with AE in the same orientation as (**a**).

On one side, AE presents a unique shallow cleft with negatively charged potential running across the surface from domain I and between domains II and III (Figure 2c). On the other side, a deep negatively charged cavity formed between domains II and III leads to a central interdomain interface and reveals a potential metal binding site (Figure 4). Interestingly, this cavity is also observed in PE but not in cholix that instead presents a pocket with positive potential (Figure 4d). No strong anomalous signal was observed for the metal ion in the electron density map, and without any obvious metal source from the crystallisation conditions, structure refinement showed a Na^+^ ion was the best fit [45]. The bound ion presents a tetrahedral coordination mediated by residues from the three different domains: E409 (domain Ib), H274 (domain II), E471 (domain III) and a water molecule stabilised by D411 (Figure 4b). Of note, E471 can also make a salt bridge with the imidazole side chain of H474 located on loop 1 of domain III and whose main chain can interact with NAD^+^ on the outskirt of the catalytic pocket. This metal-binding site is unique to AE, as only H274 is strictly conserved, but its role in the toxin function is unclear and should be assessed further. The presence of a Na^+^ ion in the centre of AE hints at a potential regulatory mechanism where the low intracellular Na^+^ concentration might affect interdomain interactions and thus access to the active site.

It should be noted that although AE was crystallised at pH 5.5, it presents an overall fold similar to the structures of PE and Cholix, which were solved at pH 7.5–8.0. This conformation may thus represent a preferred, more stable fold favoured by crystallisation of the full-length toxins. Future work should assess the impact of pH on the structure of AE and particularly its effect on the function of the toxin after activation and removal of domain I.

## 3. Conclusions

The diphtheria toxin was the first member of the mono-ADP-ribosyltransferase (mART) toxin family to be discovered and served as a model to understand the mechanism of toxicity that results in inhibition of eukaryotic protein synthesis. Although they only share limited homology with DT, PE and cholix have a similar enzymatic mechanism, and all specifically target the diphthamide of eEF2 [1]. Both DT and PE are potent virulence factors prominently involved in the pathophysiology resulting from the associated bacterial infections [46,47]. Although the role of cholix in human diseases caused by non-pandemic strains of *V. cholerae* has not been directly established, the prevalence of toxigenic strains in clinical samples and the effect of the toxin in animal models suggest it is involved in gastrointestinal infection [48]. Cholix was originally identified in genomic sequences from *V. cholerae* samples collected in aquatic environments [49] and can affect multiple animal species [5].

*Aeromonas* share similar characteristics as they are primarily found in aquatic environments, with several species emerging as serious human pathogens in a broad range of infections [17,20], as well as causing diseases in other animals [50]. Among the many virulence factors produced by *Aeromonas* [19], a *Pseudomonas* exotoxin A homologue, AE, was shown to be expressed and play a key role in the virulence of *Aeromonas hydrophila* strains causing necrotising fasciitis, likely by causing tissue damage [13,51].

Here, this study shows that AE is found in multiple *Aeromonas* pathogenic species and has all the characteristics of other diphthamide-specific mART toxins. The tri-domain crystal structure provides strong evidence that AE is a fourth member of this toxin family. The structural homology with PE in key sites of domain I suggests a similar mechanism of cell surface recognition, and future investigation should confirm if LRP1 is a main receptor. AE also has the elements required for intracellular trafficking, including the C-terminal KDEL-like signal peptide that allows retrograde transport to the ER. The exposed furin cleavage site should promote activation of the toxin in its di-chain form, which is held together by a conserved cysteine bridge that is later reduced to free the active fragment for translocation in the cytosol. Finally, domain III is the most conserved element across mART toxins, and comparison with PE indicates that the structure of AE is compatible with eEF2-binding for presentation of the diphthamide to the toxin catalytic site. The NAD^+^ binding site and catalytic pocket match the mechanism of ADP-ribosylation described previously for diphthamide-specific mART toxins. Altogether, the activity of AE is likely to result in inhibition of protein synthesis and cell death, thereby promoting bacterial infection. In view of the role of AE in *Aeromonas* infections [14], the crystal structure presented here is an important step for the development of effective anti-toxin approaches, such as mART inhibitors [52].

The unique features observed in the structure of AE are the presence of a metal-binding site in a non-conserved, negatively charged pocket centrally located between the three domains, and a clear negatively charged cleft that runs between domains I and II. The distinct electrostatic surface potential of these areas suggest that AE may be a useful tool to study the yet undefined biophysical pH-mediated modifications that occur in mART toxins. These changes are key to fully understand how the toxins subdue the host intracellular pathways to reach the cytosol of their target cells.

In addition, the discovery of AE provides a new component to the mART biotechnological toolbox. Facilitated by the toxin’s modular architecture, engineered molecules which exploits specific mART toxin functions have been developed. The cytotoxic activity of DT and PE were for example redirected for therapeutic purpose as part of immunotoxins [53]. Although these molecules were approved for the treatment of blood cancers, a limitation of immunotoxins is the rise of neutralising antibodies against the active toxin domain, which significantly hinder their efficiency over repeated usage. It has therefore been suggested that other toxins could be used to provide a substitute after the first immunotoxin neutralisation occurs [54]. AE thus represents a potential alternative to PE, although cross-reactivity to anti-toxin antibodies will need to be assessed. At the other end, the ability of domain I to promote crossing of PE and cholix through epithelial barriers has been redirected for therapeutic purpose by engineering toxin-based agents for the delivery of biopharmaceuticals [11,12]. Determining the properties and cell specificity of AE could therefore prove useful in designing novel vectors for vaccines or oral drug delivery. Overall, the crystal structure presented here offers the molecular basis to engineer alternative toxin-based biopharmaceuticals that retarget specific functions of *Aeromonas* exotoxin A for therapeutic uses.

## 4. Materials and Methods

AE expression and purification. The AE construct used in this study corresponds to a catalytically inactive mutant (E571A) [5] to prevent any safety issues. AE (NCBI WP_043170000) [residues 12–626] was codon optimised for *Escherichia coli* expression, synthesised and cloned into a pET-30 expression vector (GenScript, Piscataway, NJ, USA) with a N-terminal 6 × His-tag and TEV cleavage site. Expression was carried out in *E. coli* K12 cells (New England Biolabs, Hitchin, UK) grown in terrific broth medium at 37 °C for approximately 3 h and induced with a 1 mM final concentration of IPTG, overnight at 16 °C. Cells were harvested and frozen at −80 °C. Cell lysis for protein extraction was performed by sonication for 15 min on ice, in 0.02 M TRIS pH 8.0 with 0.2 M NaCl and 25 mM imidazole. The protein was purified by affinity chromatography (HisTrap FF, GE Healthcare, Amersham, UK), and size exclusion (Superdex200, GE Healthcare, Amersham, UK). Sample was kept at 15 mg/mL in 0.05 M MES pH 5.5 with 0.15 M NaCl, and 5% glycerol.

X-ray crystallography. Crystals of AE were grown with 1 μl of sample mixed with 1 μl of reservoir solution consisting of 12% v/v polyethylene glycol 6000, 0.1 M MES pH 5.5, 0.1 M ammonium acetate, using a hanging drop set-up. Crystals grew within 2–3 days at 16 °C. Crystals were transferred briefly into a cryo-protectant solution, consisting of the growth condition supplemented with 10% glycerol, before freezing in liquid nitrogen. Diffraction data were collected at station I03 of the Diamond Light Source (Oxon, UK), equipped with an Eiger2 XE 16M detector (Dectris, Baden, Switzerland). A Complete dataset to 2.3 Å was collected from a single crystal at 100°K. Raw data images were processed and scaled with DIALS [55] and AIMLESS [56] using the CCP4 suite 7.0 [57]. Molecular replacement was performed with the coordinates of the individual domains from PE (PDB code 1IKQ [43]) to determine initial phases for structure solution in PHASER [58]. The working models were refined using REFMAC5 [59] and manually adjusted with COOT [60]. Water molecules were added at positions where Fo−Fc electron density peaks exceeded 3σ and potential hydrogen bonds could be made. Validation was performed with MOLPROBITY [61]. Crystallographic data statistics are summarised in Table 2. The atomic coordinates and structure factors (code 6Z5H) have been deposited in the Protein Data Bank (http://wwpdb.org). Figures were drawn with PyMOL (Schrödinger, LLC, New York, NY, USA).

## Figures and Tables

**Table 1:** Structural homology of AE with other mono-ADP-ribosyl transferases (mART) toxins. Sequence and structure alignments performed with Clustal Omega and TM-align [25], respectively.

	PE		Cholix	
	% ID	rmsd (Å) [Cα]^1^	% ID	rmsd (Å) [Cα]^1^
Overall	63.6	2.5 [593]	35.4	2.9 [575]
domain Ia	54.4	1.9 [239]	33.0	2.3 [224]
domain II	58.8	1.6 [111]	33.6	2.3 [113]
domain III	71.9	1.2 [209]	45.8	1.9 [203]

^1^ Number of aligned Cα atoms.

**Table 2:** X-ray crystallography—data collection and refinement statistics.

	AE (PDB 6Z5H)
**Data collection**	
Beamline, λ (Å)	DLS-I03, 0.976
Space group	P12_1_1
Cell dimensions	
*a*, *b*, *c* (Å)	55.4, 140.2, 96.8
α, β, γ (°)	90.0, 101.8, 90.0
Resolution (Å)	2.3-56.4 (2.30-2.36)^1^
No. total/unique reflections	757,122/60,033
*R*_merge_	0.128 (0.845)^1^
*R*_pim_	0.055 (0.414)^1^
CC_1/2_	0.998 (0.895)^1^
*I/*σ*I*	12.5 (2.8)^1^
Completeness (%)	99.0 (87.2)^1^
Redundancy	12.6 (9.7)^1^
**Refinement**	
No. used reflections	56,930
*R*_work_/*R*_free_ (%)	20.4/24.7
*B*-factors (Å^2^)	
Protein (all atoms)^2^	39.5/42.3
Na^+^ ion^2^	29.0/26.9
Water	34.7
R.m.s. deviations	
Bond lengths (Å)	0.005
Bond angles (°)	1.33
Ramachandran statistics	
Favoured (%)	96.5
Outliers (%)	0.00

^1^ Values in parentheses are for highest-resolution shell. ^2^ Values for each molecule of the asymmetric unit.

## Supplementary Materials

The following are available online at https://www.mdpi.com/2072-6651/12/6/397/s1, Figure S1: Sequence alignment of Aeromonas exotoxin A from multiple *Aeromonas* species, Figure S2: Results from PSI-BLAST search of the NCBI database.
