# Supplementary material for: Crystal Structure of Exotoxin A from Aeromonas Pathogenic Species
Source: Toxins (Basel). 2020 Jun 15;12(6):397. doi: 10.3390/toxins12060397 (PMC7354439; doi:10.3390/toxins12060397)
Supplement: Supplementary file 1 [file toxins-12-00397-s001.zip › toxins-831373-supplementary.docx]

Supplementary Materials: Crystal Structure of Exotoxin A from *Aeromonas* Pathogenic Species

Geoffrey Masuyer

**Figure S1.** Sequence alignment of Aeromonas exotoxin A from multiple *Aeromonas* species.

**Figure S2.** Results from PSI-BLAST search of the NCBI database
